# Bariatric Surgery in Obesity: Effects on Gut Microbiota and Micronutrient Status

**DOI:** 10.3390/nu12010235

**Published:** 2020-01-16

**Authors:** Daniela Ciobârcă, Adriana Florinela Cătoi, Cătălin Copăescu, Doina Miere, Gianina Crișan

**Affiliations:** 1 Department of Bromatology, Hygiene, Nutrition, Faculty of Pharmacy, “Iuliu Hatieganu” University of Medicine and Pharmacy, 23 Gheorghe Marinescu Street, 400337 Cluj-Napoca, Romania; 2 Department of Physiopathology, Faculty of Medicine, “Iuliu Hatieganu” University of Medicine and Pharmacy, 3-4 Victor Babes Street, 400012 Cluj-Napoca, Romania; 3 General Surgery Department, Ponderas Hospital, 85A Nicolae G. Caramfil Street, 014142 Bucharest, Romania; catalincopaescu@gmail.com; 4 Department of Pharmaceutical Botany, Faculty of Pharmacy, “Iuliu Hatieganu” University of Medicine and Pharmacy, 23 Gheorghe Marinescu Street, 400337 Cluj-Napoca, Romania; gcrisan@umfcluj.ro

**Keywords:** bariatric surgery, obesity, gut microbiota, micronutrient deficiency, probiotics

## Abstract

Obesity is associated with reduced gut microbial diversity and a high rate of micronutrient deficiency. Bariatric surgery, the therapy of choice for severe obesity, produces sustained weight loss and improvements in obesity-related comorbidities. Also, it significantly alters the gut microbiota (GM) composition and function, which might have an important impact on the micronutrient status as GM is able to synthesize certain vitamins, such as riboflavin, folate, B_12_, or vitamin K_2_. However, recent data have reported that GM is not fully restored after bariatric surgery; therefore, manipulation of GM through probiotics represents a promising therapeutic approach in bariatric patients. In this review, we discuss the latest evidence concerning the relationship between obesity, GM and micronutrients, the impact of bariatric surgery on GM in relation with micronutrients equilibrium, and the importance of the probiotics’ supplementation in obese patients submitted to surgical treatment.

## 1. Introduction

Obesity has become a worldwide issue due to its association with increased morbidity and mortality [1]. Lately, a large body of evidence has drawn attention on the bidirectional link between obesity and GM, with obesity considered as both a cause and a consequence of GM disorder [2]. Under normal conditions, GM is involved in energy harvest, modulation of glucose and lipid homeostasis, as well as in the synthesis of vitamins [3]. A disruption of the GM balance as reported in obesity is associated with several pathogenic pathways, such as insulin resistance, chronic inflammation, and metabolic disturbances [2,3]. Moreover, regarded as a malnutrition state, obesity is accompanied by important deficiencies in vitamins and minerals, disturbances that could further impair GM and that could be augmented by the alteration in GM production of vitamins [4,5].

Severely obese patients are often hampered from losing weight and mostly from maintaining a healthy weight, and therefore reach for bariatric surgery (BS) which is, at present, the only successful treatment in these cases [6]. After BS, there is an important shift in the structure and diversity of GM [3]. Also, micronutrient deficiencies have become increasingly recognized as redoubtable late complications that may lead to severe deficiency-related disorders [7,8], among which anemia (10–74%) and neurological dysfunctions (5–9%) are most common [9]. The impaired postoperative nutritional status of the bariatric patient arises from various factors that coexist following BS: altered diet(significantly energy-restricted with higher protein intake), changes of the anatomy and physiology of the gastrointestinal tract (GIT) induced by the type of procedure performed (thus, with specific changes in digestion and absorption of food), substantial changes of the GM, and lack of adequate nutritional supplementation (6,7]. Given the significant risk to display a worsened nutritional profile after BS, these patients are in need of a rigorous follow-up and call for our attention to be focused on preventing such abnormalities, by modulating GM and proper nutritional supplementation.

The complex interplay between obesity, gut microbial population, and micronutrients, the modulation of the GM and of the micronutrients balance by BS, as well as the current knowledge and future perspectives on probiotics supplementation in morbidly obese patients that undergo BS as a therapeutic option, represent the main areas of focus within this framework.

## 2. Method for Literature Search

We conducted a systematic literature research on Pubmed, ScienceDirect, and Google Scholar, and selected publications between March 1991 and November 2019, using the search terms “obesity”, “obesity-related malnutrition”, “bariatric surgery”, “diet”, “gut microbiota”, “dysbiosis”, and “nutritional deficiencies”, individually or in combination. We included original research and review articles on both animals and humans. We also considered relevant book chapters addressing topics related to our research query. The non-English publications were excluded.

## 3. Historical Insights and Current Trends

Obesity is a chronic, relapsing disease, with a multi-factorial etiology attributable to genetic as well as environmental determinants [10]. However, none of these can fully explain the seemingly unstoppable spread of obesity worldwide [11]. Perhaps one of the most intriguing findings in the past decade of scientific research is the role that GM plays in obesity. Obesity is associated with an overall imbalance of the gut bacterial ecosystem, known as dysbiosis [12].

The era of BS developed in the 1950′s when weight loss following small bowel resections was initially observed. The refinement of surgical techniques, particularly of the minimally invasive ones, led to a dramatic increase of bariatric surgical procedures in the 2000’s [13]. However, in spite of the increasing use of BS, mechanisms underlying its effect are still incompletely understood [14].

GM is a key topic in obesity research. Between 2003 and 2017, a major expansion in the number of scientific publications investigating the GM–obesity connection was noticed. The largest number of publications indexed in the Scopus database was achieved in 2017, with a total of 702 documents [15].

Patients with morbid obesity seeking BS frequently develop nutritional deficiencies that often worsen postoperatively [9]. Recent research showed that reduced availability of micronutrients in the gut disrupts the microbial ecosystem [16]. Studies are needed to also investigate these effects in obesity-related malnutrition. Given the major anatomical and physiological changes of the GIT following BS, recently more attention has been focused on GM profile alteration following the surgical interventions. Along with diet, BS is a modulator of GM, promoting a lean host phenotype body composition [17].

One of the first pieces of evidence regarding the link between obesity and GM dates back to the 1980′s, when human studies revealed changes in the gut bacterial ecosystem following BS by using culture-dependent techniques [18]. However, at the time, our understanding of GM and its interactions with the host were extremely limited due to the small fraction of cultivable microbes. A decade later, the development of culture-independent DNA-based methods have enabled not only the phylogenetic investigation and quantification of GM, but also provided major insights into its alterations in pathological states, such as obesity. Nowadays, modern high-throughput sequencing approaches represent powerful tools for efficiently and cost-effectively surveying host–microbe complex dynamics at a level never possible before [19].

## 4. Obesity and Its Relationship with GM and Micronutrient Status

### 4.1. GM: Metabolic Functions and Modulators

The collection of all genes of the GM is termed as gut microbiome, and by these particular genes, the microbial community of the gut is involved in several physiological functions [20]. The microbiome encodes specific enzymes that have the ability to submit to fermentation certain indigestible carbohydrates and proteins which represent around 10–30% of the ingested energy. The major carbohydrate and protein fermentation end-products are short chain fatty acids (SCFAs), e.g., acetate, propionate, and butyrate, also termed as “indirect nutrients”, that are absorbed in the colon in proportion of 90–95% and that represent around 6–10% of the human energy needs [21]. The harvested SCFAs are used either as a source of energy, or as signal molecules [22].

GM is both a producer and a user of micronutrients. Microbial species that possess the ability to synthesize de novo vitamins are called prototrophs (”producers”), whereas microbial species that need exogenous provision are known as auxotrophs (”consumers”) [23]. Commensal microbial genera (e.g., *Bacteroides, Enterococcus, Bifidobacterium*) can produce most soluble B-vitamins (cobalamin, thiamine, pyridoxine, biotin, folate, nicotinic acid, panthotenic acid) and vitamin K_2._ [23,24,25]. Several microbial species possess an auxotrophic behavior, depending on microbial-derived vitamins or on other biosynthetic precursors for growth and survival. Due to this auxotrophic role, the micronutrients act as GM modulators. Nutrients acquisition occurs via de novo biosynthesis, as explained, or in the absence of biosynthetic pathways through exogenous supply from other bacteria or the host diet [16]. However, small molecule biosynthesis is a high-energy-requiring process, such that bacteria “prefer” to uptake these micronutrients from the environment whenever they are available [25]. The ability of GM to synthesize vitamins is supported by studies using germ-free (GF) animals, that have an increased need for dietary B-vitamins and vitamin K in order to maintain health [26]. However, to what extent the gut-formed vitamins impact the host’s systemic vitamins’ status is difficult to determine [27]. It should be noted that vitamins synthesized by the GM are not provisioned to the host in sufficient amounts to meet the daily nutritional needs. Moreover, inter-microbial sharing of micronutrients might decrease the bioavailability of microbial-derived vitamins for the host [26].

In adults, a healthy GM is a plastic organ that has the ability of adapting to various external and internal factors and, therefore, it is rather a changing “organ” than a stable one. Indeed, GM seems to display a set of possible configurations that can shift from one to another and by that it holds the capacity to cover the human needs and to maintain the mutualistic balanced relationship with the host. One of these triggers is diet [28]. Diet is a major factor driving rapid alterations in microbial diversity and function [28] in both a direct and indirect manner [29]. Changes in GM occur within four days of eating a specific nutrient [28]. Also, the GM responds to both weight gain/reduction nutritional strategies. Animal and human studies showed that dietary intake structure plays a fundamental role in shaping the composition of GM [30] by modulating the amount and the diversity of substrates that enable bacterial development [31].

The human GM is organized in highly complex metabolic and transcriptional networks that orchestrate diet-dependent host–microbe and microbe–microbe interactions [32]. Host–microbe associations occur in various forms, including symbiosis (mutualism, both species benefit), commensalism (one species benefits but the other is not affected), and pathogenicity (one species benefits at the expense of the other). Symbiotic/commensal interactions allow the host and/or the bacteria to utilize otherwise unavailable nutrients [31]. When gut microbes no longer engage in mutualistic relationships, dysbiosis occurs, leading to a loss of microbial diversity, expansion of pathobionts, and disease [33].

To sum up, GM is intricately involved in human metabolism and in *de novo* synthesis of certain vitamins, acting as a supplier of energy and micronutrients for the host [22].

### 4.2. Obesity and GM Dysbiosis

A milestone study came out in 2006 and revealed that obese humans display an altered GM as compared to lean controls, consisting of a reduction of Bacteroidetes and an increase of Firmicutes [34]. In an experimental model of genetically obese animals (homozygous for a mutation in the leptin gene, i.e., ob/ob mice), Ley et al. showed that cecum microbiota displayed a 50% reduction in the abundance of Bacteroidetes and a proportional increase in Firmicutes as compared to lean mice, all fed the same polysaccharide-rich diet [35]. Moreover, Turnbaugh et al. found that this “obese microbiota” is a transmissible feature by showing a significant increase in body fat of GF mice receiving the microbiota harvested from the cecum of ob/ob mice, as compared to those colonized with a GM from lean animals [36]. All these data point towards GM dysbiosis involvement in the onset of obesity.

Indeed, the existence of an interplay between GM and body weight homeostasis has been pointed out in animal studies by showing that mice harboring any microbiota from their birth contain 42% more total body fat than their GF counterparts [37]. Also, a 60% increase in body fat of GF mice that received microbiota harvested from the cecum of conventionally raised animals was observed despite a decreased food intake [36]. When disturbed, as in *ob/ob* mice, the GM displays a microbiome that has been depicted to be enriched in genes encoding several enzymes, which hold an increased ability to harvest energy from food and, therefore, pose an elevated metabolic potential that promotes a high adiposity [36].

Finally, in search of the effect of diet manipulation on GM dysbiosis and adiposity, Turnbaugh et al. submitted obese mice previously on the Western diet to two restricted diets: reduced in carbohydrates and reduced in fat, respectively [38]. The result was a significant decrease in the relative abundance of the Mollicutes and an increase of Bacteroidetes together with a reduced consumption of calories, and further with significantly less fat than the animals that continued the Western diet [38]. In a human study of obese subjects, caloric-restricted diets induced an increase in abundance of Bacteroidetes and a decrease of Firmicutes over time, irrespective of diet type (fat-restricted or carbohydrate-restricted diets) [34]. In this study, these changes were division-wide, with the bacterial diversity remaining constant over time. Interestingly, the increased abundance of Bacteroidetes correlated with the percentage of body weight loss and not with the changes in caloric intake [34].

Altogether, these data provide support to regard the dysbiotic GM and its associated genes as contributors to the onset of obesity along with an unhealthy diet, even though, as explained, in normal conditions, the involvement of GM in energy supply is small [36].

### 4.3. Micronutrient Status of BS Candidates: Impact on GM

As previously shown, between GM and micronutrients there is a double sense relationship: GM produces micronutrients, whereas micronutrients are needed for bacterial survival. Severely obese patients present various micronutrient deficiencies before undergoing BS [39,40,41,42]. The impaired nutritional status documented among the BS candidates is considered to be related to poor-quality food choices that provide insufficient amounts of vitamins and minerals in spite of a higher total caloric intake [42]. Poor eating habits characterized by low dietary diversity and intake of essential nutrients contribute to intestinal dysbiosis [43]. Other factors affecting nutritional status of patients seeking BS are related to chronic diseases [44,45], medication [46], rapid weight loss that is often required before surgery [47], or chronic dieting [48].

Several studies have observed the micronutrient deficiencies among BS candidates and reported that these patients displayed at least one vitamin or mineral baseline deficit [40,41,49,50,51]. Table 1 presents the most common nutritional deficiencies and their prevalence documented in patients evaluated before BS.

Data regarding the consequences of acute and/or chronic micronutrient shortage on the gut bacterial ecosystem is scarce. Nutrient availability shapes GM composition and plays a key role in determining the ability of the most efficient nutrient-competing bacteria to thrive. Micronutrient limitation may induce genetic and epigenetic changes, alter the main phyla, decrease bacterial richness, diversity, or gene content, facilitate overgrowth of fungi and viruses, and, finally, pave the way towards GM environment disturbances. Also, micronutrient shortage might result in blooming of pathogens and pathobionts [16,34].

Hibberd et al. showed that acute deficiency of four micronutrients, namely vitamin A, iron, folate, and zinc, impaired the GM of gnotobiotic mice harboring human bacterial species [52]. Vitamins with redox potential shape the anaerobic microbial populations in the gut due to their antioxidant defensive mechanisms, which rapidly counteract free oxygen radicals. The lack of dietary vitamin A results in a significant increase of *Bacteroides vulgatus*. Vitamin B_2_ supplementation is associated with an increase of *Faecalibacterium prausnitzii* and *Roseburia* abundance and a reduction of *Escherichia coli*, therefore decreasing the impact of oxidative stress in the gut. Shortage of vitamins C and E exert a significant inhibitory effect on Bacteroides species, while Gram-positive bacteria require more magnesium than Gram-negative bacteria [53]. Commensally anaerobes within the two major phyla, Bacteroidetes and Firmicutes, abundant in the healthy gut, are seriously reduced by the lack of dietary components with antioxidant properties (selenium, vitamins C or E), leaving room for facultative anaerobe species to flourish, such as *Escherichia coli* or pathogens *Shigella* and *Salmonella* [16].

Altogether, these data point out that micronutrient deficiencies affect not only the human host, but also its gut bacterial partners, as suggested by preclinical evidence [52]. However, a plethora of other factors might alter the diversity and function of GM and interfere with the effects induced by micronutrient shortage [16], particularly in obesity-related malnutrition.

## 5. GM and Micronutrient Deficiencies after BS

### 5.1. An Overview of BS Procedures

**Figure 1:**
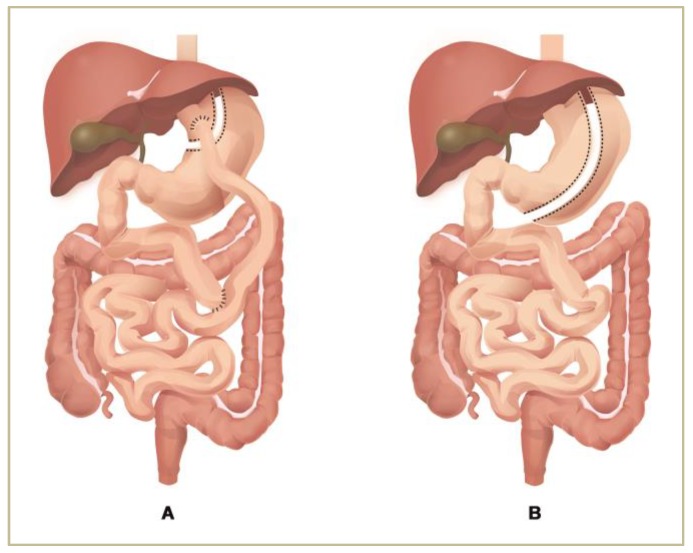
Most common BS procedures worldwide: (**A**) Roux-en-Y gastric bypass and (**B**) vertical sleeve gastrectomy.

The most commonly performed bariatric procedures worldwide are Roux-en-Y gastric bypass (RYGB) and vertical sleeve gastrectomy (VSG) [54] (Figure 1). RYGB, both a restrictive and malabsorptive bariatric technique, entails a two-step approach: first, a small tubular pouch is created in the upper part of the stomach and then the small bowel is transected 30–50 cm distal to the ligament of Treitz. The newly constructed gastric reservoir, separated from the remaining stomach, is then anastomosed to the distal end of the transected small bowel, the Roux (alimentary) limb, allowing the food to bypass the distal stomach, duodenum, and the proximal jejunum [55]. The biliopancreatic limb is connected to the alimentary limb 75–150 cm distal to the gastro-jejunostomy, creating a common channel where absorption of food occurs [56]. Thus, in addition to mechanical restriction of caloric intake, RYGB also impairs macro- and micro-nutrients’ absorption [55].

VSG involves the surgical removal of 80% of the stomach along the great curvature, including fundus, corpus, and antrum, with preservation of the pylorus, leaving a narrow gastric reservoir, called a sleeve, that has a capacity of approximately 100 mL. The decreased gastric volume restricts distention and promotes early satiety, leading to dramatically reduced portion sizes. The natural band effect exerted by the preserved pylorus also helps restriction [57]. VSG was carried out initially as the first step of a staged bariatric procedure for individuals with an extreme body mass index (BMI) (>60 kg/m^2^). After initial weight loss induced by mechanical restriction of food intake, patients underwent revision surgery to convert VSG to RYGB or to biliopancreatic diversion with duodenal switch, but soon it was noticed that VSG alone is capable of inducing substantial weight loss without the complications produced by malabsorption [57,58].

### 5.2. How does BS Change GM?

GM of extremely obese subjects submitted to BS suffers major changes in structure and function postoperatively, with a reported decrease of the Firmicutes/Bacteroidetes ratio, although the subject is still under debate [59,60].

#### 5.2.1. Taxonomical and Functional Alterations

The drastic anatomical changes of the GIT induced by BS shape the microbial landscape of the host. Taxonomical and functional alterations of the gut microbial ecosystem are believed to play a significant role in metabolic improvements experienced following BS [61]. Changes in glucose homeostasis after RYGB and VSG that occur well before any significant weight loss is achieved are mostly mediated by weight-independent mechanisms [14]. Causal evidence linking GM to surgery-induced weight loss is highlighted by animal and human fecal transplants from patients submitted to BS to mice raised without any exposure to microorganisms. In a landmark study from 2015, Tremaroli et al. analyzed the long-term effects of BS on GM by colonizing GF mice with fecal samples collected from humans nine years after RYGB or vertical banded gastroplasty, and also from obese controls [59]. Two weeks after transplantation, mice receiving microbiota from BS patients gained less fat mass (46% and 26%, respectively) compared to mice colonized with microbial communities from obese patients. Microbial alterations following BS typically occur within three months postoperatively [61], and even as early as seven days after RYGB, in both murine models and humans [14].

Studies using various sequencing methods reported high microbial gene richness (MGR) and diversity of gut microbial populations after RYGB and VSG [62,63,64] and a shift from “obese” to a “lesser obese” microbial structure [65]. Low MGR, exhibited by 75% of individuals with severe obesity [63], is associated with increased BMI, inflammation, and insulin resistance [62]. However, despite dramatic weight loss and improvement of metabolic markers observed after weight loss surgery, MGR is not fully corrected at one year after RYGB and it remains similar after five years postoperatively [63]. This finding challenges the presumed role of BS-related GM alterations in modulating beneficial postoperative metabolic effects [66]. It is noteworthy that surgical treatment alone cannot account for improved MGR seen after BS, as other mechanisms (i.e., metabolic and inflammatory amelioration, weight loss, or diet) are thought to also contribute to this outcome. Indeed, short-term energy-restricted dietary intervention was shown to improve MGR in obese/overweight patients [67] and also to increase phylogenetic diversity in obese patients with type 2 diabetes [68]. Moreover, it is hypothesized that late weight regain and reappearance of obesity-related comorbidities documented in some bariatric patients might be linked with the lack of complete restoration of GM following BS [62].

The taxonomic composition of the gut microbial population is significantly impacted following weight loss surgery (Table 2). The most common change reported by the majority of animal [69] and human studies [30,70,71,72,73] is a relative decrease in the abundance of Firmicutes and an increase in Bacteroidetes, Proteobacteria, and its class Gammaproteobacteria (order Enterobacteriales, family Enterobacteriaceae, genus *Escherichia*). Notably, the GM profile differs significantly between rodents and humans [14]. Proteobacteria proliferates following BS due to increased luminal pH and high levels of dissolved oxygen that enables the growth of facultative aerobic microorganisms and inhibits anaerobic populations [74]. Reduced gastric volume following surgery increases pH levels of the stomach and distal gut, leading to changes in resident bacterial populations and microbial overgrowth. Overall, decreased gastrointestinal acidity favors the presence of *Akkermansia muciniphila, E.coli,* and *Bacteroides spp*. or bacteria species typically associated with the oral microbiota [61]. Greater microbial diversity reported postoperatively also includes an increase in the phyla Verrucomicrobia and Fusobacteria, and decreased proportion of Actinobacteria [60,75].

There are different microbiota-related outcomes in relation to different surgical procedures. However, there are few studies exploring these outcomes. Medina et al. reported results consistent with previous research in a study comparing the impact of different surgical treatments on GM, namelyan important abundance in Proteobacteria following both RYGB and VSG [74]. Bacteroidetes’ proportions increased after RYGB, but decreased after VSG, despite similar weight loss at six months postoperatively. A single common compositional change, i.e., an increase in *Roseburia* abundance, was reported by Murphy et al. following RYGB and VSG in patients with type 2 diabetes remission [74]. Differential changes in GM composition were reported after RYGB and VSG also in murine models of BS. Shao et al. showed that RYGB led to significantly lower body weight than VSG nine weeks postoperatively and also produced a greater shift on GM compared to VSG [76]. These results are explained by different rearrangements of the digestive tract associated with RYGB. This technique alters the GIT and intestinal environment to a greater extent and consequently exhibits stronger effects on GM composition compared to VSG.

Functional alterations of GM following BS were also explored by a few small-scale studies [62,73,77]. Recent data showed that surgery-related taxonomical changes shaped the functional capacity of microbial populations mostly in the first three months postoperatively [78] and this effect was sustained up to nine years [59]. According to KEGG (Kyoto Encyclopedia of Genes and Genomes) orthologs and pathway analysis, BS led to changes in gut microbial metabolism regarding essential nutrient transport, such as thiamine, vitamin B_12_, manganese, iron, and zinc, carbohydrate utilization (transport system for monosaccharides, phosphotransferaze systems), amino acids uptake (putrescine, lysine/arginine/ornitine, histidine, glutamate transportation) purine or fatty acids metabolism (beta oxidation) [59,78,79]. Murphy et al. reported that 1 year following surgery RYGB was associated with more substantial functional changes of GM compared to VSG, despite similar diet, weight loss, or remission of type 2 diabetes [79]. A rapid change in the functional capacity of GM was reported 3 months following VSG in 23 obese patients [64]. Decreases in microbial functions, such as pathways related to carbohydrate fermentation, citrate cycle, glycosaminoglycan degradation, and lipopolisaccharides synthesis, were observed after surgical treatment as microbial enzymatic activity became more similar to that of lean controls. Three months post-VSG, a marked increase of *B. thetaiotaomicron* comparable to that of lean controls was documented. Increased levels of *B. thetaiotaomicron* were inversely associated with BMI and circulating glutamate levels. Anti-obesogenic effects of *B. thetaiotaomicron* are modulated by the mutualistic interactions with other bacterial species [64].

#### 5.2.2. The Impact of BS-Related Diet Change and Weight Loss on GM

The specific mechanisms underlying GM modifications provided by BS have not yet been elucidated. As previously explained, diet is considered to be one of the major factors impacting GM composition and function. An individual′s microbiota composition remains relatively stable over time if no dietary shifts occur [29], but this is clearly not the case for extremely obese patients submitted to RYGB or VSG whose post-surgery food intake suffers major quantitative and qualitative alterations. Energy intake of bariatric patients is 40–50% lower six months postoperatively [80], with an average decrease of 1800 kilocalories/day compared to prior surgery intake. Due to transient intolerance of protein-rich foods, in the first year following BS the actual protein intake is 0.5 g/kg/day and does not meet the recommended daily allowances for bariatric patients of 1.5 g/kg/day. Fat and carbohydrate intake are also diminished during the first year postoperatively. Lower glycemic index carbohydrates are often preferred to high glycemic index carbohydrates [81]. Caloric restriction alters bacterial community structure in a matter of days to weeks depending on an individual′s MGR [29].

Alterations of gut microbial ecology are also driven by surgical weight loss or rearrangement of the GIT following BS [2]. Whether diet dominates over weight modification following BS in shaping GM [12] or if GM change is a direct consequence of BS *per se* are still matters of debate. Paganelli et al. assessed short-term temporal changes in GM composition and diversity, before submission to a 2 week crash diet (baseline), by the end of this trial, and 1 week, 3 months, and 6 months postoperatively, in 45 patients undergoing RYGB (*n* = 23) and VSG (*n* = 22). Interestingly, results showed that, unlike the crash diet that induced a significant but temporary shift in GM, BS itself was responsible for more persistent alterations in bacterial composition as well as for restoration of microbial diversity, which enabled the surgery-related weight loss. Regarding the relative abundance and beta-diversity of gut bacteria, no significant differences were reported between patients undergoing either RYGB or VSG prior to surgery, after 1 week or after 6 months postoperatively. Patients experienced similar weight loss after both interventions, which might explain the same changes observed in GM composition, irrespective of surgery type [82].

Severe dietary restriction and impaired absorption following BS requires life-long vitamin and mineral supplementation. Hence, the greater post-surgical capacity of enteric microbes to uptake and utilize micronutrients might be due to their increased bioavailability [78]. On the other hand, absorption of substrates could be substantially enhanced by restricted diet in order to compensate for host energetic and nutritional needs [72,81].

### 5.3. Nutritional Significance of Small Intestine Bacterial Overgrowth

Obese patients submitted to BS may develop small intestine bacterial overgrowth (SIBO), a condition that can itself interfere with the weight loss process or increase the risk of micronutrient deficiencies. A recent prospective study including 378 patients with morbid obesity showed that SIBO was present in 15% of patients before RYGB and increased up to 40% after surgery [83].

Bacterial overgrowth develops in the context of an intestinal stasis, which allows coliform microorganisms’ proliferation in the small bowel. Mechanical stasis is a frequent cause of intestinal stasis associated with gastrointestinal surgery, including RYGB, and creation of blind loops. Although the small intestine normally hosts some bacterial populations, it is the type and the number of these microorganisms that cause the symptoms of SIBO. Bacteria recognized as SIBO resemble bacteria normally found in the colon, respectively gram-negative aerobes and anaerobes species, such as *Escherichia coli, Enterococcus* spp., *Klebsiella pneumonia*, or *Proteus mirabilis*, that metabolize undigested carbohydrates into SCFAs and gas. Excessive growth enables the atypical bacteria in the proximal small bowel to compete for nutrients with the human host, whereas its metabolites can cause mucosal injury [84].

SIBO is mostly defined in a quantitative manner as greater than 105 bacteria (colony-forming units)/mL of proximal jejunal aspiration [84]. However, SIBO diagnosis based on the small bowel aspirate test is invasive and costly for clinical practice. Alternatively, breath tests are used more often (H_2_ or methane), although they yield an inferior diagnostic accuracy compared to the small intestine aspirate test [85]. The most practical method is the “therapeutic trial”, that recommends administration of treatment when clinical manifestations associated with SIBO occur [86].

SIBO is associated with various gastrointestinal symptoms, from bloating and diarrhea to nutrients’ malabsorption, depending on the specific type of colonic bacteria that hyperproliferates in the small bowel [87]. The inflammatory response following the luminal overgrowth of atypical microbes elicits changes of the epithelial cells that cause villous atrophy and/or stimulate the synthesis of inflammatory cytokines leading to an impaired absorptive capacity of macro- and micro-nutrients. Fat and fat-soluble vitamins’ (particularly A, E, and D) malabsorption occurs because of improper micelle formation due to bacterial deconjugation of bile acids [84,88]. However, vitamin K levels are generally normal or elevated in patients with SIBO since bacteria are able to synthesize menaquinone [89]. As a result of bile acids’ deconjugation carried out by small bowel bacteria, lithocholic acid is formed, a metabolite with potent toxic properties that further exacerbates the intestinal epithelial cell dysfunction and also contributes to carbohydrate and protein malabsorption [84]. Impaired carbohydrate uptake develops due to both decreased brush border enzyme activity and availability of substrate, because small bowel bacteria prematurely metabolize it. Increased amounts of small intestine microorganisms also compete with the host for intraluminal protein, which affects absorption of amino acids and peptides. Moreover, in SIBO patients, a decreased level of enterokinases that lead to impaired proteolytic reactions and subsequently to disturbed activation of pancreatic zymogens was also observed [89,90].

Studies support the concept that SIBO also impairs absorption of thiamine and B_12_ vitamin. Lakhani et al. showed, in a retrospective analysis of 80 patients submitted to RYGB, that thiamine deficiency measured as whole-blood thiamine diphosphate level was lower than the reference range in 39 patients [91]. Of these patients, 28 presented elevated folate plasma levels, a marker suggesting the presence of SIBO [88], and 15 were also diagnosed with SIBO by undergoing glucose-hydrogen breath testing. Persistent thiamine deficiency rapidly resolved after initiation of antibiotic therapy to treat SIBO [91]. Improper uptake of B_12_ vitamin was described in the presence of SIBO due to bacterial synthesis of biologically inactive B_12_ analogues [86]. Impaired B_12_ absorption following RYGB leads to the onset of secondary megaloblastic anemia. Machado et al. showed, in a case report of two patients submitted to RYGB which tested positive for SIBO postoperatively, that although antibiotic treatment improved hemoglobin levels, mean cell volume was still increased while B_12_ level was below the normal range [88]. 

Establishing causal relationships between surgery-related factors and alterations in GM composition and function following BS is difficult, since there are major metabolic and hormonal changes occurring concomitantly in the early postoperative state [92]. Besides the anatomical rearrangement of the gut, weight loss, and diet, other factors are also involved in the sustained changes of the GM after BS, such as biliary acids and hormones [75].

### 5.4. Pathogenesis of Micronutrient Deficiencies after BS

**Figure 2:**
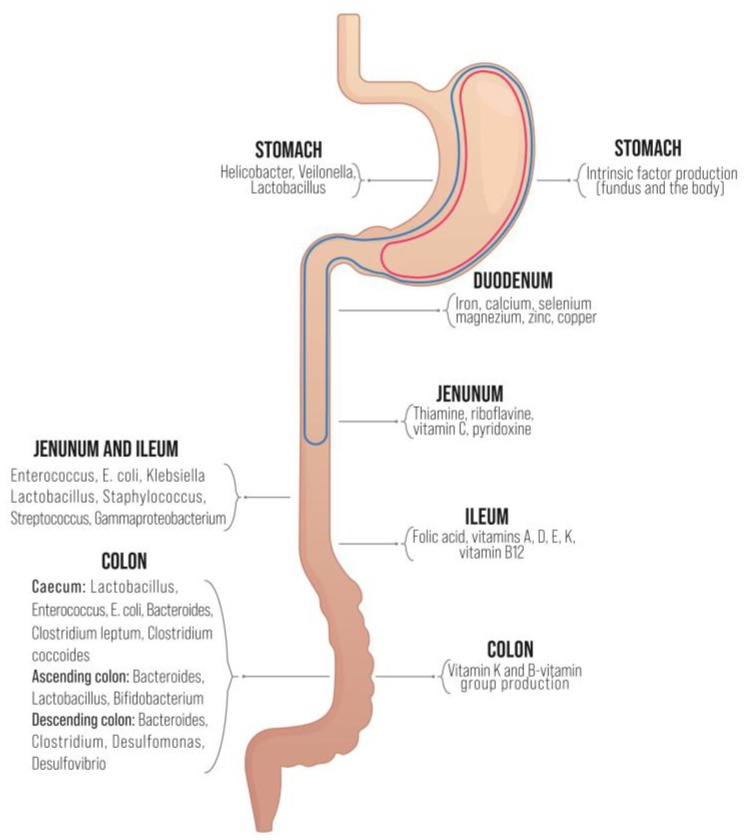
Distribution of micronutrient absorption/biosynthesis sites within the gut [94] and the associated microbiota [95]. Marked areas are excluded after RYGB (blue) and VSG (red).

Micronutrient deficiencies resulting from BS may lead to serious postoperative nutritional and metabolic complications. Whereas most of these can be predicted and corrected preoperatively, certain deficits often persist following BS, in spite of vitamin and mineral supplementation. Micronutrient-related complications of BS require more clinical attention to enable early discovery and appropriate treatment [93]. A representation of the major sites of vitamins’ and minerals’ absorption and microbial communities’ distribution across the GIT is illustrated in Figure 2.

**Figure 3:**
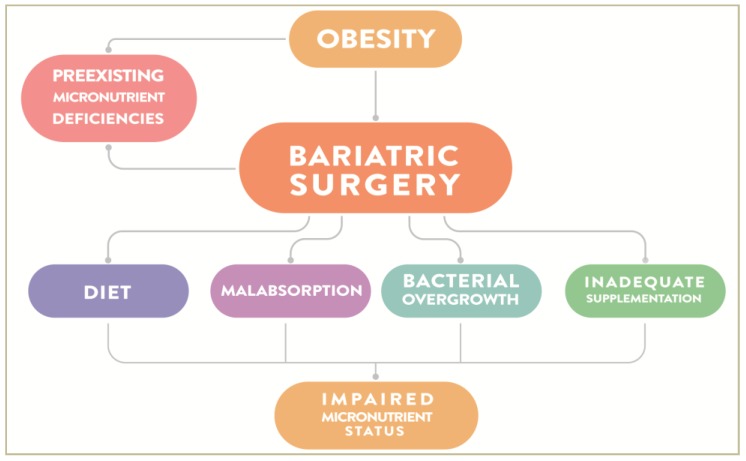
Schematic diagram illustrating the main factors responsible for micronutrient deficiencies in bariatric patients after surgery. Baseline nutritional shortage may worsen postoperative deficiencies. Changes in eating behavior, decreased absorptive capacity, small intestine bacterial overgrowth, and poor compliance to postoperative dietary optimization and nutritional supplementation also contribute to the state of micronutrient deficiency following BS.

Micronutrient deficiencies are frequently reported after RYGB and VSG [9,51,96,97], with an incidence as great as 50% in mid- and long-term follow-ups [86]. The underlying variables associated to postoperative vitamin and mineral deficiencies are both surgery- and patient-related (Figure 3) [98].

Postoperative restriction of food intake, reduced appetite, and changes in gastrointestinal hormone profile are common weight loss mechanisms, well-documented after both RYGB and VSG, that also affect nutrient status [99]. However, the onset of surgery-related micronutrient deficiencies secondary to RYGB and VSG are explained by different factors. The malabsorptive component of RYGB affects vitamins’ and minerals’ absorption due to exclusion of the remnant stomach and the upper part of the small intestine from the gastrointestinal transit. Nutrients cannot be absorbed in the alimentary and biliopancreatic limbs since food bolus is not exposed to biliopancreatic secretions. However, the degree of malabsorption is related to the length of the common channel (distal jejunum, ileum, and colon) rather than the lengths of the Roux limb [100]. A decreased absorptive capacity in the common portion of the small intestine may also occur as a result of asynergia between food bolus, bile acids, and pancreatic enzymes. Micronutrient absorption (especially vitamin B_12_) following RYGB is also impaired due to a lower output of gastric juice as a result of bypassing the distal stomach [101].

Since the small bowel remains intact after surgery, one would expect fewer micronutrient deficiencies to be reported after VSG compared to RYGB [98,101,102]. However, several recent studies showed that micronutrient deficiencies may occur to a similar degree following both surgical techniques in spite of an intact intestinal absorptive surface area in VSG [103,104]. VSG affects the micronutrient status by changing gastrointestinal motility, i.e., accelerating gastric emptying and gastro-duodenal transit time, as well as reducing hydrochloric acid and intrinsic factor secretion. Due to the gastric fundus resection and hypochlorhydria, liberation and dissolution of certain vitamins and minerals might be impaired [102,104].

Other surgical-related variables that lead to vitamin and mineral deficiencies are consequences of post-surgery complications, such as recurrent nausea and vomiting, food intolerances, or SIBO that was previously discussed [105]. Patient-related variables that impact the postoperative micronutrient status of individuals submitted to BS refer to substance and alcohol abuse and to poor compliance to nutritional recommendations and supplementation protocol. RYGB is associated with a greater risk for the occurrence of alcohol use disorders due to modified alcohol pharmakokinetics, yielding a higher peak blood alcohol concentration that is achieved faster compared to non-operated controls, “addiction transfer” (i.e., from food to alcohol), and an increased alcohol reward mediated by a neurobiological mechanism, as shown in animal models. A prospective study conducted by King et al. that followed more than 2000 weight loss surgery patients at ten hospitals across the United States for up to seven years reported that 20% of patients submitted to RYGB developed alcohol use disorder [106]. In a 2019 questionnaire-based survey on 533 BS patients, Mahawar et al. showed that slightly over half of the respondents reported non-adherence to micronutrient supplementation [107]. The main micronutrient deficiencies reported after RYGB and VSG include vitamin B_12_, folic acid, iron, thiamine (vitamin B_1_), vitamin D, and calcium [108,109,110,111,112]. Other reports on nutritional deficiencies after weight loss surgery, particularly following mixed bariatric procedures, are for fat liposoluble soluble vitamins, namely, vitamin A [113], vitamin E [114], and vitamin K [115], as well as for copper [116], zinc, and selenium [117,118].

#### 5.4.1. Vitamin B_12_

Vitamin B_12_ (also called cobalamin) is a water-soluble vitamin required as a coenzyme by two metabolically important enzymes: methionine synthase, responsible for methylation of homocysteine to methionine, and metylmalonyl CoA-mutase, which catalyzes reversible isomerization of L-metylmaolnyl-CoA to succinyl-CoA. Since biosynthesis of B_12_ is a complex 30-step process limited to prokaryotes, humans require a dietary source of this molecule. Intrinsic factor (IF), synthesized by the parietal cells of the stomach, plays a major role in cobalamin absorption [119].

Changes in the architecture of the GIT secondary to gastric fundus resection result in decreased secretion of both HCl and pepsin by the functional remnant segment. This prevents capture of vitamin B_12_ from dietary sources and determines loss of food contact with intrinsic factor (IF)-producing cells, leading to cobalamin malabsorption and deficiency [120,121]. Disrupted IF secretion is currently considered the main driver of the post-surgical B_12_ deficiency [121,122]. A 2017 small-scale study, involving 20 patients submitted to RYGB, proposed that, besides IF, other molecules involved in the vitamin B_12_ metabolism may be involved in the pathogenesis of its postoperative deficiency. The authors reported a decrease in gastric production of transcobalamin 1 (TCN_1_) after RYGB that affects B_12_ intestinal transport. Cobalamin preferentially binds to TCN_1_ in the low acidic gastric environment and attaches to IF only in the small upper bowel. Using transcriptomic analysis, increased B_12_-receptor encoding genes’ expression (CUBN) was detected at all levels of the GIT, suggesting a potential genetic reprogramming of the intestinal tissue in order to compensate for insufficient B_12_ delivery [121]. In spite of indirect improvement of cobalamin absorption following mixed bariatric procedures, studies show a greater incidence of vitamin B_12_ deficit after RYGB compared to VSG [123]. Vitamin B_12_ deficiency is reported in 37–50% of gastric bypass patients [122] and, respectively, in 10–20% of patients that underwent VSG [104]. The authors argue that after restrictive BS, although TCN_1_ synthesis may be inhibited, exposure of food at the entire intestinal surface area results in local IF production, with B_12_ gut transport by IF being less disrupted [121].

B_12_ deficiency is associated with a triad of symptoms known as Biermer’s disease: megaloblastic anemia, gastrointestinal, and neurologic symptoms [124]. Kornerup et al. recently showed that, in spite of supplementation with physiological doses, B_12_ status becomes impaired within a few months following BS because of decreased absorptive capacity. Hence, administration of high doses of B_12_ is recommended to be initiated right after surgery [125].

#### 5.4.2. Folic Acid

Folate absorption occurs primarily in the upper small intestine (proximal jejunum), requiring conversion of dietary folates into absorbable units and acidic milieu at the cell surface [126]. Folate deficiency becomes evident in tissues with rapidly replicating cells, altering both cell division and protein synthesis, and resulting in megaloblastic anemia and megaloblastic bone marrow changes. Since short-term inadequate dietary intake is quickly mirrored in serum folate levels (after 3 weeks), its measurement is not a reliable index of folate status. Tissue stores are more accurately reflected by red blood cell folate, whose levels decline after 3 to 4 months of inadequate intake and are not influenced by diet [127,128].

Folate deficiency ranges between 9% and 39% following both malabsorptive and restrictive procedures [7], being related to poor eating habits [129]. Folate deficiency can be elicited following BS due to the depletion of tissue stores as a result of inadequate dietary intake, as well as impaired absorption due to hypochlorhydria and altered intestinal pH [127]. Post-surgery deficiency of folate cycle cofactors—vitamin B_6_, vitamin B_12_, and folate—is associated with increased plasma levels of homocysteine [129]. However, recent research has shown that folate may be synthetized by colonic bacteria and seems to be absorbed throughout the entire small intestine and colon, with a lowering absorption gradient from jejunum to colon. Therefore, postoperative supplementation following RYGB with physiologic doses (400 mcg) suffices to prevent or correct the folate deficiency due to compensatory intestinal absorptive capacity [130].

#### 5.4.3. Vitamin B_1_

Vitamin B_1_, also known as thiamine, plays a crucial role in three enzymatic systems that require its active form, thiamine pyrophosphate (TPP), as a cofactor. Thiamine-dependent enzymes are involved in energy metabolism, biosynthesis of nucleic acids, or antioxidant defense mechanisms. The brain depends upon mitochondrial ATP production and therefore is highly sensitive to thiamine shortages [131]. Low levels of intracellular thiamine are associated with energy failure and increased production of oxygen reactive species. Since the body’s pool of thiamine of about 30 g represents only 30 times the daily requirements, symptoms of deficiency rapidly develop when food intake fails to meet the nutritional needs. Body stores become depleted after only 20 days of inadequate oral intake and thiamine deficiency occurs faster than for any other vitamins [132,133].

A broad range of pathological conditions is associated with thiamine deficiency, including beriberi, neuropathy, and acute or chronic encephalopathies, such as Wernicke’s encephalopathy (WE) or Korsakoff syndrome [134]. WE is a medical emergency and is characterized by sudden onset of nystagmus, ataxia, ophthalmoplegia, and altered mental state [135]. Thiamine deficiency generally develops in bariatric patients within 6 months following surgery, mostly due to hyperemesis. Out of 118 cases of WE following RYGB and VSG, almost 90% confirmed hyperemesis as a risk factor [136]. Intractable vomiting impairs absorption of thiamine and so deficiency can occur despite oral supplementation. Therefore, early diagnosis of thiamine deficiency is crucial to prevent permanent sequelae, such as ataxia or impaired ocular motility and mental status [134]. However, in one third of the cases, initial symptoms of WE are often not recognized, leading to further complications [136].

Long-term parenteral nutrition, excessive alcohol intake, poor appetite, upper small bowel exclusion or bacterial overgrowth also affect thiamine status of bariatric patients [91,133,137]. Although symptoms of thiamine deficiency are well described after malabsorptive procedures, its prevalence in bariatric patients cannot be precisely estimated [110,137]. Clements et al. observed a biochemical thiamine deficiency occurrence rate in 18% of patients 2 years after RYGB [138], in agreement with later findings of Lakhani et al., which reported the same level of clinical thiamine deficiency after surgical follow-up [91]. In a recent retrospective study, Tang et al. reported decreased levels of thiamine in 25.7% of patients within 1 year after VSG [136]. Long-term alterations in thiamine serum concentration were also observed in 30.8% of VSG patients after 5 years, despite routine supplementation [139].

#### 5.4.4. Vitamin D and Calcium

Absorption of vitamin D occurs mostly in the jejunum and ileum through passive diffusion, a mechanism which rather requires the presence of bile salts than fat [140]. After mixed bariatric procedures, gastric, pancreatic, and biliary secretions travel through the biliopancreatic limb in an undiluted state and only blend with the nutrients in the distal jejunum. Therefore, less contact between fat, fat-soluble molecules, and bile exists before food bolus passes to the ileum [141]. Following VSG, vitamin D malabsorption might result from less exposure of the nutrients to the digestive mucosa [142]. Hypovitaminosis D following BS can also be precipitated by SIBO [105].

Chronic vitamin D deficiency leads to metabolic bone disease (MBD), a condition linked to gastrointestinal surgery, particularly gastrectomy [140]. Both reduced bone mineral density and bone remodeling have been observed 3 years after RYGB and, in a lesser degree, after VSG. However, postoperative skeletal consequences may not be primarily caused by disrupted absorption of vitamin D and calcium [143]. In bariatric patients, the risk of developing MBD following surgery remains increased for the rest of their lives. Both RYGB and VSG impair dissolution and solubilization of nutrients due to poor acid secretion [144].

RYGB is associated with direct calcium malabsorption, irrespective of vitamin D levels, due to bypassing the upper small bowel, the site of active calcium transport. Hypochlorhydria caused by surgical procedures and medical therapy with proton pump inhibitors also affect calcium uptake. However, if vitamin D status and calcium intake are adequate, a severe decline in calcium absorption capacity following RYGB is not necessarily expected, as paracellular transport functions throughout the length of the small intestine [144] and to a significantly lower extent in the colon [145]. Studies using the dual stable isotope method showed that after RYGB intestinal absorption, calcium is significantly affected over short- (6 months) [146], medium- (12 months), or long-term (24 months) postoperatively [147]. Noteworthy, in the short-term study, calcium uptake was severely diminished in spite of adequate calcium intake or optimized levels of vitamin D [146].

Several potential mechanisms might alter calcium uptake in postoperative VSG patients, such as vitamin D deficiency, reduced caloric intake, hypochlorhidria, or proton pump inhibitors’ administration [144]. Medium- and long-term alterations in calcium absorption were reported in pre-menopausal women following surgery, i.e., VSG, although this technique does not involve changes of intestinal anatomy [147]. In the largest cohort to date (999 subjects), postoperative hypocalcemia prevalence was reported in 3.6% of patients, 1.9% of patients (*n* = 15) underwent RYGB, and 9.3% (*n* = 13) of patients were submitted to VSG. The lowest calcium concentration was observed after 1200 days in the RYGB group respectively, after 239 days in the VSG group. Recommended daily calcium intake provided through both diet and supplements varied between 1500 and 2000 mg [148].

Vitamin D deficiency and impaired absorption of calcium in the gut contribute to bone loss because of secondary hyperparathyroidism (sPTH) [142]. Elevated levels of PTH are associated with bone calcium resorption, higher vitamin D synthesis, osteomalacia, or loss of bone mineral density (BMD) that cause hip and column osteoporotic fractures [149]. As early as 3 months following RYGB, calcium malabsorption and sPTH develop and bone turnover increases. Decreased hip BMD has been reported in 8–11% of patients at 1 year after RYGB, whereas only small reductions of BMD have been observed in the spine. Adverse skeletal effects following VSG have not been so well documented. However, bone loss has been observed 6 months following this bariatric procedure [150].

#### 5.4.5. Iron

After ingestion, the gastric acidic environment enhances iron (Fe) absorption by maintaining its solubility and converting it from the ferric state (3^+^) to the ferrous form (2^+^), the only form of iron that can be absorbed in the GIT [104].

Several mechanisms underlie the pathogenesis of postsurgical iron deficiency. Iron bioavailability and absorption are affected by both dietary and physiological factors [120]. Diminished HCl secretion in the gastric pouch as well as decreased intestinal absorptive surface (duodenum and proximal jejunum) [151] and administration of H_2_ blockers or proton pump inhibitors significantly impair iron absorption. Also, iron-rich food intake after BS is considerably decreased due to both caloric restriction and food aversions, especially to red meat [120]. In a 2014 study including 72 individuals submitted to RYGB, Nicoletti et al. reported that red meat intolerance occurred in 49.2%, 42.2%, 46.4%, and 39% of patients after 1, 2, 3, and 4 years postoperatively [152]. In addition, after RYGB, the most efficient area for iron absorption—the duodenum—is bypassed [104]. Iron deficiency following VSG is determined by the malabsorption secondary to the gastric resection that prevents reduction of Fe^3+^ to Fe^2+^ [151]. Iron deficiency is very common after BS and has been reported in 18–53% of patients after RYGB and respectively, in 1–53% patients after VSG [153].

Iron deficiency results in mycrocytic anemia [120], which is not easy to diagnose or treat following BS since it mimics other mineral deficiencies [154]. Vitamin B_12_ and folate deficiencies can also coexist. Therapeutic management of iron deficiency anemia consists of administration of oral or parenteral iron compounds. Given the high rate of gastrointestinal side effects, impaired absorption, and poor adherence to oral supplementation scheme, intravenous iron preparations are mostly prescribed [151]. Regular monitoring of iron status through laboratory assessment and preventive post-surgical supplementation are recommended [104].

#### 5.4.6. Other Micronutrient Deficiencies

- *Fat Soluble Vitamins*

Perturbations of fat soluble vitamins’ serum levels (vitamin A, E, and K) are associated with malabsorptive bariatric procedures [7,101,155]. However, the frequency of these nutritional deficiencies following weight loss surgery is generally low and clinical manifestations are rarely reported [101,156].

Vitamin A deficiency is induced by iatrogenic malabsorption and severely diminished retinol and carotenoids’ intake due to calorie restriction. Moreover, a specific diet following weight loss surgery provides low levels of fat that limit vitamin A uptake. Non-alcoholic steatohepatitis and cirrhosis, frequently observed in bariatric patients, might impede vitamin A storage and synthesis following surgery [157]. Prevalence of vitamin A deficiency following RYGB ranges between 8% and 11% [86]. In a recent study, Brandão et al. observed no effects on serum vitamin A concentration or visual function following both RYGB or VSG [158].

Few data are available regarding vitamin E and K deficiency after BS [7,86]. In a 2014 survey, Cuesta et al. observed vitamin E deficiency to be present in 8.7% of patients 1 year following RYGB, after adjustment of serum levels to total cholesterol [159]. Reported vitamin E deficiency prevalence in bariatric literature after this type of surgical procedure varies between 0% and 22% [77]. A recent systematic review concluded that, although rare following weight loss surgery, symptomatic vitamin K deficiency may occur in patients submitted to major malabsorptive procedures. Consequently, these patients should be closely monitored after their surgical treatment [160].

- *Zinc and Copper*

Zinc absorption proceeds in the small bowel through a specific active carrier mechanism that, under normal physiological conditions, is not saturated. If the oral zinc supply increases (e.g., supplementation following BS) [161], zinc uptake also increases up to a maximum level. A diet low in zinc enhances its intestinal absorption, whereas high intakes allow zinc transport through a passive, paracellular pathway [162]. In the small bowel, zinc competes with both iron and copper for absorption. Hence, bariatric patients are advised to administer these supplements separately. If zinc and copper are taken together, they should be supplemented in an appropriate ratio [161]. Gehrer et al. observed a slightly higher post-surgical zinc deficiency 1 year following RYGB (37%) than VSG (34%) [102]. Another study analyzing micronutrient deficiencies after RYGB and VSG with a 5 year follow-up indicated that serum zinc concentrations were reduced in 25.7% and 12.5% of patients, respectively [163]. Zinc deficiency after RYGB is generally asymptomatic and varies between 8% and 25.7% long-term, when routinely supplemented [161].

Copper is an essential micronutrient and crucial cofactor for various enzymes involved in a plethora of processes, including hemoglobin synthesis, nervous system functioning, or cellular respiration [164]. Prevalence of copper deficiency after RYGB is approximately 10%. Like zinc, the risk of developing symptomatic hypocupremia after surgery is rare among patients who adhere to prescribed nutritional supplementation [165]. Pellitero et al. observed a tendency for copper concentration to decline during the first two postoperative years in patients submitted to VSG. The authors found 9.8% of patients to be deficient in copper 5 years after surgery [166].

- *Selenium*

Selenium is an important trace element and antioxidant in the form of selenocysteine, the 21st amino acid used in human protein synthesis [167]. According to Papamargaritis et al., serum levels of zinc, copper, and selenium were found to be relatively stable after both RYGB and VSG in supplemented patients, although a likelihood for selenium serum levels to decrease was observed [168]. Selenium deficiency was not documented by Pellitero et al. in VSG patients following surgery [166].

Dietary sources and postoperative recommended supplementation of micronutrients in order to prevent deficiencies after RYGB and VSG are detailed in Table 3.

Micronutrient status of bariatric patients tends to further deteriorate after BS, which, in turn, affects the configuration and composition of GM [16,52]. As explained, since low MGR is not completely reversed after BS [62], specific interventions aimed at further restoring the correct microbial balance and improving GM–host interactions are needed after the surgical treatment [63]. One of these strategies is probiotic therapy, which will be further discussed. Administration of probiotics appears to be associated not only with greater weight loss in bariatric patients, but also with decreased SIBO and, thus, with improved vitamin synthesis and availability, leading to an optimized micronutrient status [181].

## 6. Probiotics and GM: Implications for Bariatric Patients

Therapeutic interventions designed to reshape GM structure have gained increasing attention in the last years as they have been found to mitigate the risk/degree of obesity [182]. Modulation of the host GM is achieved through diet and/or administration of probiotics [183].

Lactic acid bacteria (LAB) are normally present in both human GM and fermented food [184,185]. LAB have long been used as probiotics to prevent or treat disease in humans [186] due to the safety of the therapy, the lack of reported side effects, and appropriate long-term administration [187], as shown in human trials [188]. Bacterial strains belonging to *Lactobacillus, Bifidobacterium,* and *Sacharomyces* genera are most commonly employed as probiotics [186]. Contrary to some beliefs, probiotics bring benefits to the host even in the absence of colonizing the gut or inducing major changes in GM [189]. It should be noted that the high-pH environment following RYGB ensures better survival for probiotic bacteria during passage through the otherwise harsh acidic environment of the GIT, making patients submitted to this surgical procedure a promising target population for probiotic therapy [182].

Development of faster and efficient genetic sequencing tools as well as understanding of GM composition and function expanded the list of bacterial strains associated with beneficial effects for human health [190]. Among these, *Akkermansia muciniphila, Faecalibacterium prausnitzii,* and members of *Clostridium* clusters IV, XIVa, and XVIII have emerged as next-generation probiotics [33]. In a 2019 proof-of-concept study, supplementation of 10^10^ *Akkermansia muciniphila* to obese and overweight volunteers improved certain metabolic parameters [191]. *Akkermansia muciniphila,* a member of Verrucomicrobia phylum, is severely decreased in obesity and other metabolic disturbances [33].

Human studies on the efficacy of conventional probiotics after BS is limited, although the usage of probiotics is common postoperatively [192]. To date, the effects of probiotics following weight loss surgery have been analyzed by 4 studies, out of which, 3 were conducted on patients with obesity who underwent RYGB and 1 was performed among patients submitted to VSG [181,193,194]. The latter did not report any improvements in hepatic, inflammatory, or other clinical endpoints associated with probiotic therapy at 6 months, respectively 1 year following surgery, suggesting that VSG in itself results in major metabolic changes, leaving little or no room for an extra effect [192]. On the other hand, Chen et al. observed alleviation of gastrointestinal symptoms and improvement in quality of life in 60 RYGB patients two weeks after the initiation of probiotics and digestive enzymes therapy [193]. A triple-blind randomized controlled trial conducted on 9 RYGB patients showed that prebiotics supplementation alone, not synbiotics, increased weight loss [194]. Probiotic administration was associated with improvements of SIBO and increased weight loss in 44 patients following RYGB. Woodard et al. also reported significantly increased levels of vitamin B_12_ in the probiotic group at 3 and 6 months postoperatively that might correlate with the reduced bacterial overgrowth [181]. This finding is extremely important as weight loss surgery is associated with an increased risk for B_12_ deficiency and suggests that probiotic supplementation might improve micronutrient status of bariatric patients postoperatively [181].

An increasing body of evidence demonstrates that certain strains of LAB and *Bifidobacteria* naturally occurring in human gut or used as starters in fermented products are able to synthesize B-group vitamins (including folates, riboflavin, vitamin B_12_) as gut commensals [24,185]. *L. fermentum CECT5716,* a human breast milk strain, is recognized as a B_2_ and B_9_ producer, although no clinical trial investigated its impact on plasma vitamin concentration after administration. *L. rhamnosus GG,* that inhabit the gut of healthy humans, is the only probiotic strain whose thiamine-producing ability was demonstrated. *Bifidobacterum lactis BB12* encodes all the genes for thiamine production, although its synthetic capacity has not yet been confirmed [185]. *B. adolescentis* and *B. pseudocatenulatum* are folate-producing probiotic strains associated with increased concentration of folate in human feces, suggesting that folate synthetized in the gut is absorbed and used by the host. Increased levels of folate in colon, cecum, and plasma were observed in folate-deficient rats following a diet rich in bifidogenic components, which imply that folate-producing probiotics might function as a vitamin supplier and prevent an in situ state of deficiency [195].

The use of probiotic bacteria able to provide exposure to natural vitamins may represent a valuable alternative to enrichment of food with synthetic molecules [196] and may also facilitate the development of GM-targeted therapies aimed at restoring micronutrient equilibrium.

## 7. Conclusions

Obesity is associated with the impairment of the GM and micronutrient deficiencies. BS-mediated weight loss and/or improvement or remission of obesity-related comorbidities following surgical treatment are associated with significant changes in GM composition and function. These favorable changes of gut bacterial landscape might impact micronutrient production and availability. However, BS seems to not be able to fully restore microbial balance postoperatively. Moreover, there are some deleterious consequences that reside from the onset of SIBO, which affect both the weight loss process and the micronutrient status of the patients submitted to BS.

It is difficult to point towards the exact factor that induces the GM changes after BS, namely diet, weight loss, and surgery itself. Although the impact of surgery is still not clear and the changes observed following the surgical treatment for obesity are not consistent between studies, GM modification after BS should be considered in the context of restricted energy intake and altered dietary quality. Also, it seems that there is no difference with regard to GM modulation between the two most currently performed weight loss surgery techniques, i.e., RYGB and VSG.

Besides the lack of complete rescue of GM and the installation of SIBO following BS, the presence of preoperative micronutrient deficiencies as well as the failure of proper supplementation after surgery result in postoperative micronutrients deficiencies. Therefore, the administration of probiotics seems to be an adequate means that might render the host more balanced in terms of GM and micronutrients milieu.

Further understanding of how gut microbes impact the host ability to digest and absorb nutrients, how bacteria acquire vitamins, and how they adapt their metabolism and regulate gene expression in a micronutrient-poor environment may be of high interest for future research. Identification and developing of novel probiotics may contribute to a more effective clinical approach for the management of postoperative micronutrient deficiencies and for optimizing GM evolution following weight loss surgery.

## Figures and Tables

**Table 1:** Micronutrient deficiencies prior to bariatric surgery (BS) [40].

Micronutrient	Prevalence
**Vitamin D**	65–93%
**Iron**	13–47%
**Vitamin B_12_**	4–13%
**Folate**	0–32%

**Table 2:** Changes of human GM composition following BS [73].

↑/↓	RYGB	VSG
↑	*Akkermansia* (Verrucomicrobia)	*Bulleidia* (Firmicutes)
↑	*Escherichia* (Proteobacteria)	*Roseburia intestinalis* (Firmicutes)
↑	*Klebsiella* (Proteobacteria)	*Faecalibacterium prausnitzii* (Firmicutes)
↓	*Lactobacillus* (Firmicutes)	*Coprococcus comes* (Firmicutes)
↓	*Bifidobacterium* (Actinobacteria)	
↓	*Faecalibacterium prausnitzii* (Firmicutes)	
↓	*Coprococcus comes* (Firmicutes)	

↑—increased; ↓—decreased.

**Table 3:** Micronutrient deficiencies following RYGB and VSG.

Micronutrient	Food Sources	Recommended Supplementation after BS	References
**Vitamin B_12_**	Eggs, milk, cheese, red meat, poultry, fish, liver, fortified soy or cereals	350–500 mcg daily (sublingual/liquid) or 1000 mcg monthly (parenteral)	[169,170]
**Folic acid**	Liver, green leafy vegetables	400–800 mcg daily	[170,171]
**Vitamin B_1_**	Pork, poultry, whole-grains, brown rice, soybeans, nuts, dried beans, peas, fortified cereals	12 mg daily/50 mg dose from B-complex supplement/multivitamin twice daily	[170,172]
**Vitamin D**	Dairy, fatty fish (salmon, sardines, mackerel), egg, offal	3000 IU daily until plasma concentration exceeds 30 ng/mol	[170,173]
**Calcium**	Dairy, green leafy vegetables, fruits	1200–1500 mg/day	[170,174]
**Iron**	Meat, fish, legumes, lentils, soybeans, green leafy vegetables, cereals, breads, spinach, turnip	18 mg daily (multivitamin)	[170,175]
**Vitamin A**	Eggs (yolk), liver, dairy products, fish, red/orange/yellow fruits	5000–10,000 IU/day	[170,176]
**Vitamin E**	Vegetable oil, seeds, fruits, vegetables	15 mg/day	[170,177]
**Vitamin K**	Green leafy vegetables (broccoli, collards, spinach)	90–120 ug/day	[170,178]
**Zinc**	Oysters, beef, pork, veal, lamb	8–11 mg/day	[170,179]
**Copper**	Organ meats, nuts and seeds, chocolate, shellfish	1 mg/day	
**Selenium**	Cereals, meats, fish	-	[180]

RYGB—Roux-en-y gastric bypass; VSG—vertical sleeve gastrectomy.
